# Know Thyself, Improve Thyself: Personalized LLMs for Self-Knowledge and Moral Enhancement

**DOI:** 10.1007/s11948-024-00518-9

**Published:** 2024-11-21

**Authors:** Alberto Giubilini, Sebastian Porsdam Mann, Cristina Voinea, Brian Earp, Julian Savulescu

**Affiliations:** 1 Uehiro Oxford Institute and Wellcome Centre for Ethics and Humanities, University of Oxford, Oxford, UK; 2 Center for Advanced Studies in Bioscience Innovation Law (CeBIL), Faculty of Law, University of Copenhagen, Copenhagen, Denmark; 3 Centre for Biomedical Ethics, Yong Loo Lin School of Medicine, National University of Singapore, Singapore, Singapore

**Keywords:** Moral AI, Self-knowledge, Authenticity

## Abstract

In this paper, we suggest that *personalized* LLMs trained on information written by or otherwise pertaining to an individual could serve as artificial moral advisors (AMAs) that account for the dynamic nature of personal morality. These LLM-based AMAs would harness users’ past and present data to infer and make explicit their sometimes-shifting values and preferences, thereby fostering self-knowledge. Further, these systems may also assist in processes of self-creation, by helping users reflect on the kind of person they want to be and the actions and goals necessary for so becoming. The feasibility of LLMs providing such personalized moral insights remains uncertain pending further technical development. Nevertheless, we argue that this approach addresses limitations in existing AMA proposals reliant on either predetermined values or introspective self-knowledge.

## Introduction

AI systems increasingly mediate major areas of life. Large Language Models (LLMs) are used for customer support, image and text generation, financial guidance, and political strategy formulation, among other tasks. Increasingly, LLM applications also impact our affective lives by recognizing, eliciting, and simulating emotions. For instance, AI is used in psychiatry in various ways, including chatbots for talk therapy, virtual reality for the treatment of post-traumatic stress disorder and prediction of suicidal behaviour (Minerva & Giubilini, 2023).

Perhaps not surprisingly, the idea of developing AI life assistants, capable of handling various professional and personal tasks, including offering tailored life advice, has caught the attention of artists and companies. In a recent Park Theatre production, *Disruption*, a wealthy investor pitches “an algorithm that is more complex than the human brain and promises to guide them through big life decisions better and more effectively than they can guide themselves.” But it’s not just fiction, as Google is in the process of creating a “life coach”, with engineers “testing the assistant’s ability to answer intimate questions about challenges in people’s lives.” (Grant, 2023).

There is already a growing body of literature exploring how AI systems should be designed to be artificial moral advisors (AMAs) that enhance moral decision-making. These proposals span a spectrum, from AI systems assisting human moral decision-making (Giubilini & Savulescu, 2018; Savulescu & Maslen, 2015), whether via robotic nudges (Borenstein & Arkin, 2016; Klincewicz, 2019) or Socratic questioning (Lara & Deckers, 2020), to complete delegation of moral decisions to AI (due to perceived lack of bias and access to extensive data) (Dietrich, 2011; Whitby, 2011; Hubbard & Greenblum, 2020)).

As we argue, these proposals share a common weakness: they assume personal morality is static. On this view, the task of AMAs is to help individuals either to clarify what their moral values are or how to live according to them. Yet, as we suggest, while some moral values may be relatively stable over the medium term, context and events can modify the expression or weight given to them. Other values may themselves change as a function of experience.

In this paper, we suggest that *personalized* LLMs trained on information written by or otherwise pertaining to an individual could serve as AMAs that account for the dynamic nature of personal morality. The LLM-based AMAs proposed in this paper would harness users’ past and present data to infer and make explicit their sometimes-shifting values and preferences, thereby fostering self-knowledge. Further, these systems may also assist in processes of self-creation, by helping users reflect on the kind of person they want to be and the actions and goals necessary for so becoming. The feasibility of LLMs providing such personalized moral insights remains uncertain pending further technical development. Nevertheless, we argue this approach addresses limitations in existing AMA proposals reliant on either predetermined values or introspective self-knowledge.

## Knowing Oneself and One’s Moral Values: Ontological and Epistemological Considerations About the Self

In this section, we briefly explore several philosophical theories of self-knowledge, addressing both ontological aspects regarding how the self is constituted,and epistemological questions regarding how we come to know it. We argue that at least part of our self-knowledge derives from inferring our values from experience. In the following section, we show how adequately trained LLMs can contribute to our self-knowledge by assisting in inferentialist processes of self-knowledge.

“Know thyself” is an ancient injunction. For our purposes, self-knowledge can be defined as a state of awareness of the presence or absence of beliefs, values, or desires within oneself that are reason-giving. It therefore enables individuals to understand the motivations behind their conduct and evaluate their choices in light of other beliefs and values they hold—or aspire to hold (Bortolotti & Mameli, 2006). Conceptually and psychologically, knowing oneself is closely related to knowing one’s moral values as they are expressed in one’s attitudes, beliefs, and behaviours (Strohminger & Nichols, 2014, 2015).

There are two aspects of the relationship between values and self-knowledge relevant for this paper. First, some of our values emerge from our choices, so understanding oneself involves recognizing what these values are. Second, our values and moral choices express our identity and give us an indication of who we really are. In what follows, we argue that these two aspects reveal that while our sense of self may be relatively stable in the short to medium term, it is influenced by changing contexts and decisions over longer periods. We suggest that properly trained LLMs, by helping us understand what are the values that derive from our experiences, could assist us in understanding our selves. Before looking at how they can do that, we will consider in more detail the relationship between values and self-knowledge.

Firstly, it might not always make sense to talk of our ‘true’ moral values that exist before and independently of our decisions. This is because the context in which we make such decisions shapes and adapts our values (Liu et al., 2022, p. 438). Among other things, our experiences, the feedback we receive from others, and other variables related to the specific context within which we find ourselves affect our moral and political views (Kalmoe et al., 2020).

Philosophical views which see the self as something that is created through action and defined by the choices we make, rather than the other way around, lend support to this idea: “existence precedes essence”, as Jean-Paul Sartre (1973) famously claimed. Most of us do not have a well worked out system of moral beliefs, or an ethical theory, or a ranking of values that we simply apply to our decision making. Even according to Aristotle, virtue is something that is created through behaviour, rather than something that precedes it and is applied to behaviour to make it moral: “we become just by doing just actions, temperate by temperate actions, and courageous by courageous actions” (Aristotle, Nichomachean Ethics, 2000 ed.). More recently, evidence from moral psychology suggests that even our so-called ‘moral foundations’, often taken to be fundamental dispositions shaping or even determining our moral and political views (Haidt, 2012; Haidt & Graham, 2007), are subject to significant change over time based on our experience. The causal links with our moral and political ideologies are bidirectional: our moral foundations are influenced by our changing moral or political views as much as they influence them (Smith et al., 2017). This shows that we do not always apply the same well-defined moral values to real world situations.

Second, our moral choices are not only about what we think is right or wrong, but also about behaving in a manner that is consistent with who we are or aspire to be. That is, they are about being authentic, or true to one’s self. The importance for each of us of living an authentic life cannot be underestimated. It is quite telling, for instance, that psychiatric patients often prioritize authenticity over mere wellbeing when making decisions about taking drugs to treat mental disorders (Erler & Hope, 2014). Authenticity can mean different things (Erler, 2014), but on any understanding of it, to live authentically requires, at least to some degree, knowing oneself. Lynch (2005) brings out the value of self-knowledge to authenticity by suggesting that someone who does not know what matters to them lacks control over themselves. Oftentimes we have conflicting desires, preferences and even values, and not knowing which ones we identify with can stop us from living authentically. Thus, self-knowledge is constitutive of authenticity.

For example, suppose you are an environmentalist who cares deeply about reducing your carbon footprint. Yet you are also deeply committed to your family, who live far away. You have the resources to visit them via air travel but know that this would significantly increase your environmental impact. Should you travel? Many moral choices are like that: they involve not only a weighing of values, but also an assessment of which values are most important to one’s sense of self. You want to do the right thing, but you also want to be true to yourself. But again, what is that self, and how do you acquire that type of knowledge?

Answering these questions is difficult precisely because often we don’t know what our values are before we make the choices that either express or shape them. The expectation that our choices reveal and shape our values creates a circular dynamic, making the process of self-knowledge a difficult task. Some external assistance, from outside of this loop, might be helpful.

Different traditions of thought have been divided over whether our true self is primarily a matter of self-discovery (e.g. Taylor, 1991) or self-creation (e.g. Frankfurt, 1988, De Grazia, 2005). Roughly speaking, on the first view, our authentic self is something relatively stable (Taylor, 1991)—which reflects the Romantic ideal of an inner nature that we are called to realize. On the second view, the true self is created by endorsing certain desires, preferences, and values we hold through the choices we make, at the expense of conflicting ones—which reflects the existentialist idea of taking responsibility for who we are (Sartre, 1973).

These philosophical models are not mutually exclusive, as they might simply be taken to illuminate different aspects of the concept of the self. Indeed, some of the views that we have briefly sketched above are more nuanced than the dichotomy between self-discovery vs self-creation suggests (Erler, 2014), making space for elements of self-discovery in models that are largely based on self-creation (De Grazia, 2005), and vice versa (Taylor, 1991). A synthesis that seems plausible to us is that a relatively stable self exists which guides our choices, but this self evolves over time rather than being innate or fixed. While stable in the near or medium term, over longer periods this self is shaped by changing contexts and the choices made therein.

The two different understandings of the self and authenticity comprise both an ontological view of the nature of the self and an epistemological view about our knowledge of this self. The former concerns whether there is a stable entity that is expressed in our choices or something dynamic that our choices constantly shape. The second concerns whether self-knowledge is achieved through introspection or inferentially by observing our behaviour.

Ontological and epistemological view are not completely independent of one another. While an ontology of the self as self-creation is compatible with an epistemology of introspection, it also provides supports for epistemological views according to which self-knowledge is oftentimes acquired by inferring our values and preferences from our past experience, rather than being discovered by introspection (Cassam, 2014). On this view, we build our self-knowledge—primarily or at least in part—inferentially from various types of evidence about ourselves, rather than by introspection as suggested by traditional Cartesian models. The evidence-base for such inferences can include own past choices and “internal promptings” (Lawlor, 2009, pp. 48–49), such as our feelings or imaginings (Cassam, 2014). Some of the evidence base for such inferences is available to others as well, and indeed sometimes others have access to this evidence in ways that we do not. For instance, we might adopt patterns of behaviour that we do not recognize, but that our close friends do.

It is not our purpose to settle the complex philosophical and psychological questions of whether the inferentialist or introspective accounts of self-knowledge are correct, or how they might coexist. While introspection can play an important role in self-knowledge, we don’t need to deny its value to recognize that inferring what our values are from our behavior can also be a valid method of self-knowledge. To the extent that such inferentialist view captures at least something true about how we get to know our self and our values, the AMA model we present below would constitute an improvement over current proposals.

## Procedural Moral Assistants

The close connection between our own moral values and our sense of the self provides a helpful conceptual tool to see why many proposals for artificial assistants have been criticized both on philosophical and psychological grounds. They focus on telling right from wrong on the basis of a given set of values, whether provided by human users (Giubilini & Savulescu, 2018; Savulescu & Maslen, 2015) or pre-packed in the algorithm (Borenstein & Atkin, 2016). However, they have disregarded the fact that one’s self and the set of values one can identify with, or at least part of them, are frequently changing and often not explicitly held or recognized.

For ease of presentation, we adopt a classification provided by Lara and Deckers (2020) in which they group extant proposals into three categories: “exhaustive enhancement”; “auxiliary enhancement”; and their own “Socratic assistant”. AMAs in the first category seek to supplant or supplement human moral decision-making by providing fully formed judgments for action, either to be implemented directly (Dietrich, 2001) or offered as suggestions for human action (Gips, 1995). These proposals assume moral values and reasoning can be encoded in algorithms that lack human biases. However, by outsourcing moral deliberation entirely to machines, they both remove chances for humans to develop their moral reasoning abilities and fail to account for change in human values over time and context. If morality is shaped by one's experiences over time, then moral advisors cannot rely on fixed sets of data and procedures; they must be flexible and adapt to users' evolving values.

The second category, auxiliary enhancement, includes proposals for AMAs that provide guidance based on moral values explicitly specified by the user. For example, Giubilini and Savulescu (2018) and Savulescu and Maslen (2015) propose AI assistants that give advice according to the moral principles chosen by the user. AMAs in this category would enable humans to be better moral agents by providing morally relevant empirical information, according to the moral values that humans choose, which would be difficult for humans to gather by themselves. Even if users can update and adjust the values they provide, the advisor itself works based on static inputs rather than dynamically modelling users' evolving values. Thus, while auxiliary AMAs allow for some personalization, they still lack the flexibility to fully capture the contextual development of morality over time. Their guidance may become misaligned as users' values shift.

Neither of these two models is adequate to capture the dynamic nature of our values as delineated in the previous section, whereby our experience and choices constantly reshape and create our values, rather than being based on a stable set of values.

The third category of AMAs analysed by Lara and Decker is meant to address this aspect (Lara & Decker, 2020; Lara, 2021; developing a proposal by Seville & Field, 2011): a “Socratic” virtual assistant that would interrogate the user about their moral values, resembling a Socratic process. The Socratic assistant would respond to the user’s prompts by making judgements about their “empirical, conceptual, logical-argumentative and ethical rigour” (Lara, 2021, p. 10). This would put the user in the best condition possible for engaging in rational deliberation.

Building on Lara and Deckers, we discuss two further proposals under this category. First, Klincewicz's AI nudger (2020) employs Stoic strategies such as "assessment of control" and "imagining calamity" to help users cultivate a rational and emotionally controlled disposition, thereby guiding them toward making ethical choices that are congruent with Stoic philosophy. Second, Borenstein and Arkin's (2016) robotic nudging proposal aims to create "socially just tendencies" by using verbal and bodily cues to nudge human behaviours into alignment with John Rawls' theory of justice, specifically focusing on the reinforcement or discouragement of certain types of behaviours.

While these models offer innovative approaches to moral guidance, they too fail to fully account for the dynamic nature of moral values. Klincewicz's AI nudger and Borenstein and Arkin's robotic nudging are tethered to specific frameworks—Stoic practices and Rawlsian justice, respectively—which may not fit or adapt to the evolving moral values of individual users. The Socratic assistant assesses users' responses based on criteria such as "empirical, conceptual, logical-argumentative and ethical rigour," which, although important for fostering informed moral reasoning, may nevertheless subtly align with specific metaethical stances. For instance, the emphasis on logical-argumentative rigour may implicitly favour cognitivist metaethical views, thus limiting the scope and role of emotions in moral reasoning.

In summary, AMA proposals to date either remove moral decision-making entirely from humans, offer guidance based on static sets of moral values, or operate on the assumption that improving introspective capacities necessarily enhances, or is the only way to enhance, moral deliberation. These presuppositions entail an epistemological commitment to the primacy of first-person knowledge as well as ontological commitments to a pre-existing, innate self with stable moral values. However, there are both philosophical and empirical reasons to question this view. Philosophical accounts of authenticity problematize the notions of an unchanging, innate self and stable set of values, while psychological evidence indicate that our moral preferences and the weight we assign them are context-dependent (Liu et al., 2022). Thus, models proposed to date at best only capture the introspective part of the potential of AMAs to lead to greater self-knowledge and to moral enhancement. In what follows, we propose that the missing inferential element can be supplied by a more sophisticated algorithmic approach in the form of LLMs fine-tuned on person-specific information. Such *personalized* LLMs, we argue, could infer third-person knowledge about the self inferentially from past behaviour.

## LLMs and Personalised Moral Advisors

Advances in LLMs (Bommasini et al., 2022; Bubeck et al., 2023), we suggest, hold significant potential for the development of more adaptive AMAs. A particularly promising approach is the use of personalized LLMs fine-tuned on or otherwise adapted to individual-specific information. Personalized LLMs have been piloted (Porsdam Mann et al., 2023) or proposed (Earp et al., 2024) for various person-specific applications, such as academic prose generation (‘AUTOGEN’) or personalised patient preference prediction (‘P4’) in the case of medical decision-making incapacity, respectively. Here we suggest that a LLM could likewise be personalised on information produced by, describing, or otherwise pertaining to an individual, for the purposes of providing the kind of third-party, inferential and longitudinal knowledge about one’s self described above.

We propose a personalised LLM for self-knowledge and moral guidance: the *iSAGE* (individualized System for Applied Guidance in Ethics). This hypothetical iSAGE would use fine-tuned LLMs to serve as a 'digital ethical twin,' resembling the idea of a 'digital psychological twin' for healthcare preferences as discussed by de Kerckhove (2021): a real-time consultative interface that helps individuals make morally sound decisions based on their own evolving set of values and beliefs.

The iSAGE would rely on a corpus of text directly or indirectly related to an individual's ethical views and decision-making history. This corpus would be chosen by the individual themselves as part of the iSAGE configuration process. To address privacy concerns, the iSAGE could operate on locally stored LLMs (as opposed to uploaded to the internet). The text used to train iSAGE could include, but is not limited to, written reflections on ethical topics, social media interactions, responses to ethical questionnaires, and transcripts of conversations about moral dilemmas (see Earp et al., 2024, for a related proposal). The model's training data could extend beyond textual contributions to include behavioural metrics, as values and preferences can sometimes be inferred from behaviour (think, for instance, how platforms like Amazon or Facebook infer your preferences from your online behaviour, such as which videos you watch or links you click on), or speech recordings. The objective is to obtain a well-rounded profile of a person's moral stance and general character, which would then be used for fine-tuning the LLM.

A fundamental assumption of this proposal is that LLMs in general, and the iSAGE in particular, would be able to infer values and preferences based on writings and descriptions concerning an individual. This assumption is supported by recent empirical evidence demonstrating related abilities in LLMs. Studies have shown that LLMs can effectively model human preferences and adapt to user-specific profiles in the context of risk and investment advice (Kim et al., 2024). Namikoshi et al. (2024) demonstrated that fine-tuning LLMs improves their ability to model the preferences of targeted populations. Dong et al. (2024) found that LLMs can accurately judge user preferences based on personas when allowed to report their confidence, achieving over 80% accuracy on high-confidence predictions and sometimes outperforming human evaluators. State-of-the-art LLMs achieve adult-level performance on higher-order theory of mind tasks (Street et al., 2024). Existing research confirms LLMs' capabilities to adapt to user-specific political profiles (Simmons, 2023), encode moral preferences (Schrerrer et al., 2023), and to capture societal biases (Bender et al., 2021). Furthermore, specialized AI research is committed to aligning these systems with human values, both generally and individually (Askell et al., 2021; Gabriel, 2020; Kenton et al., 2021; Kirk et al., 2024), while emerging studies indicate LLMs' proficiency in generating consensus statements (Bakker et al., 2022).

Though this evidence demonstrates that LLMs possess capacities that align with the conceptual requirements of iSAGE, that is, inferring values and preferences from person- or group-specific data, it is important to acknowledge that these have not yet been applied to or tested in the context of providing personalized self-knowledge or moral guidance. Several practical and technical issues will require addressing before a system like iSAGE could be implemented. These include ensuring that the training corpora used are of a sufficient volume and quality to reflect individuals’ ethical views and character as well as properly addressing data security and privacy issues. We consider these issues in more detail after we describe how we envision iSAGE working in practice.

## The iSAGE in Practice

The role played by technology in how people understand themselves and the world around is increasingly gaining attention (Leuenberger, 2023; Postan, 2016). For instance, Leuenberger (2023) explores the influence of personal information technologies on our narrative identity. Specifically, Leuenberger shows that while personal information technologies can offer new insights for our self-narrative, they can also downplay the importance of certain life events and facets of our identity, thereby altering what we take as important in defining ourselves.

Our proposal shifts the focus away from how today’s technology impacts self-perception and identity, pointing towards how we should design it so that it fosters self-knowledge and self-creation. iSAGE centres on the idea of using an individual’s historical and current data to infer and explicitly express their occasionally changing values and preferences, thus helping with the acquisition of self-knowledge. Moreover, iSAGE can also help with self-creation by assisting users in reflecting on their aspirational selves and using the collective human knowledge of LLMs.

The iSAGE as described would have the following advantages. Firstly, to the extent that some moral values and preferences are relatively stable over short- or medium timeframes, a system like iSAGE would provide a further means beyond introspection to gain insight into these values and preferences. This self-insight function would be operationalised in interactions between the user and the iSAGE in which a user asks the system for advice or insight. A response might take the following form: “the kind of person who made the choices that you have made so far [or who wrote these types of thoughts about these types of circumstances, etc.], is the kind of person who prioritizes value *x* and value *y* over value *z* and would therefore likely make decision *a* instead of decision *b* in the current situation.”

Importantly, as we envision it, iSAGE would not only output evaluations but also qualify and explain the degree of confidence it has in its output, giving its ‘reasoning’ (i.e., the factors it has relied upon, and the weights given to these factors, in determining its evaluation). This would enable users to judge and evaluate the model’s output in cases where that output differs from a user’s own introspective assessment. Users can thus either accept iSAGE’s evaluation or they can reject it. But even in the latter case they would be prompted to think of whether they adhere or not to a certain characterization, which can foster acquisition of self-knowledge (see Demaree-Cotton et al., 2022; Porsdam Mann et al., 2024).

Secondly, to the extent that moral values and preferences evolve over time, iSAGE would analyse patterns in a user's history to model their changing priorities. Plentiful psychological evidence suggests that the accuracy of one’s memory of the past is inhibited by cognitive biases and distortions. Examples include the egocentric (Krueger & Clement, 1994) and choice-supportive biases (Zorn et al., 2020), in which individuals interpret the past generally or their past choices specifically in a self-serving manner (see also Carlsen et al., 2020); and the euphoric recall and negativity biases (Unkelbach et al., 2020), which lead to certain positive or negative aspects, respectively, of the past to be given disproportionate salience in memories. These tendencies could be counteracted by a system like iSAGE with access to documented evidence of past behaviour and preferences as expressed in its training data.

Thirdly, and relatedly, given sufficient data the iSAGE could extrapolate from a user’s past and present values, preferences, and character to predict their evolution into the future. Though highly speculative, such an extension of iSAGE’s temporal personality and preference insights from retrospection to prospection would have all kinds of interesting implications for iSAGE applications like moral enhancement and character development. The system could forecast how a user's personality and priorities may change in response to major life events they have not yet experienced but can be anticipated. This information could then be used for advanced planning and present decision-making.

Fourthly, iSAGE can highlight divergences between a user's lived conduct versus their stated and/or inferred values. For instance, if a user voices support for climate action but behaves in carbon-intensive ways, iSAGE can surface this apparent discrepancy for reflection. This assists users in resolving conflicts between ideals and actions. Bringing awareness to these divergences can motivate change in either ideals or actions to restore congruence and reduce hypocrisy and/or cognitive dissonance. Sometimes those around us act as a mirror of our identity through their reactions, whether by describing how they see us or evoking memories of our history (Leuenberger, 2023, p. 14). iSAGE too could act as a personalized ‘moral mirror’ in which one’s self-image is reflected against a third-person perspective. Such a moral mirror could assist in the hard work of gradually reconciling self-image with deeds through critical self-evaluation and intentional self-development over time.

Fifthly, as a fine-tuned LLM, the iSAGE would retain many of the technical capacities of its underlying model. This means it could integrate with various other sources of information as well as other applications in addition to leveraging general LLM affordances and abilities. This is relevant in several ways. By connecting iSAGE to ongoing sources of data, such as biometric sensors (i.e., fitness trackers), productivity suites including email, financial data, and social media accounts, the quality and volume of training data could be significantly increased and continually updated. Third-party applications or plug-ins such as WolframAlpha as well as user-assembled custom knowledge bases could provide iSAGE with reliable information which it could use to make its advice more realistic. Like other LLMs, iSAGE can use its natural language processing abilities to customize its outputs to the method and style of communication that best suits a particular user, similar to Zohny’s (2023) proposed use of LLMs for personalised knowledge consumption. Using software like LangChain, iSAGE could also be connected to other LLMs in an ensemble or committee approach to moral guidance (see Chang, 2024 for a similar proposal).

Consider a user named Alex who cares deeply about family and loyalty. Alex makes time for weekly calls with her parents and visits every holiday. However, Alex's new job requires long hours and frequent business travel, taking time away from family. By interfacing with Alex's calendar and email, iSAGE can track this growing divergence between Alex's family values and conduct. It can point to the increasing intervals between visits and suggest scheduling video calls before upcoming trips.

Leveraging its integrative data, iSAGE can inferentially highlight this emerging value-action gap to Alex for self-reflection, providing a third-person perspective Alex may lack the introspective self-awareness to notice. Further, by analysing longitudinal patterns, iSAGE can note that family and loyalty have become less dominant values for Alex relative to career and success over the past year. Making this evolution explicit allows Alex to evaluate whether it aligns with intentions for character development. Moreover, iSAGE could show Alex how her character can change in the future, allowing her to take necessary measures to fasten or slow down such a development and see whether it fits the ideal projection of the type of person she would like to be.

Lastly, iSAGE could leverage other individuals’ personalized LLMs. For example, iSAGE could be merged with or consult a model based on someone considered a moral “role model” for advice. An individual could pick their favourite moral or political philosopher. Instead of the outdated “Ask Jeeves” one could “Ask Peter Singer” or “Ask Michael Sandel”, or “Ask Jurgen Habermas”. Already LLMs have been trained on philosophers’ work with convincing responses based on the philosopher’s actual views (see Schwitzgebel et al., 2023, on a digital version of philosopher Daniel Dennett). This would import desired moral expertise and potentially broaden the individual’s own moral horizons, while also engaging that individual’s values and past experience and life.

## Conclusion. The Place of iSAGE?

As we see it, the iSAGE can complement or supplant other AMAs by incorporating an inferentialist or constructivist model of individual moral preferences and values. The iSAGE’s output would contain advice on a given situation but crucially also provide reasoning for the advice given. Thus, the iSAGE is meant as a tool to spark reflection and action as to the relationship between our values and preferences, our aspirational self, and our past experience and choices. If implemented properly, the iSAGE could help individuals construct good lives by helping them explore who they have been, who they are, and who they want to become. In this way, it could contribute to the moral enhancement and character development of individuals, thus potentially benefitting society at large.

As our contribution is theoretical only, much work remains to be done, for example in determining which types of input best capture moral values and preferences, how outputs should best be structured to reflect advice and the reasoning leading to that advice, including its degree of uncertainty, how users interpret and react to such outputs, as well as numerous more technical questions. Especially important are issues surrounding consent and privacy. While we envision use of iSAGE or similar systems to be an entirely voluntary endeavour, it is nevertheless important that the user provides clear and informed consent to the types of data to be used during its configuration process. Since the type of information required is potentially highly sensitive, privacy issues need to be adequately addressed. One way to do so would be to use dedicated LLMs (rather than LLMs by general providers also used for many other purposes) hosted in physically and digitally secure environments. Another would be to use lightweight LLMs that can be stored locally. This is already possible at the time of writing for desktop computers and some laptops (e.g., GPT4All) and projects are emerging which may be able to run on mobile phones and other handheld devices (e.g., MLC LLM).

Leuenberger (2023) points out some of the problems that personal information technologies can raise when they become tools for identity co-narration. Some of these concerns are also relevant to the case of iSAGE. The image created by iSAGE can leave users unsure of themselves and insecure of their capacities of portraying themselves correctly, which could lead to a sort of moral deskilling. Think of a situation where someone considers themselves an environmentalist, but iSAGE shows that despite their efforts, they did not manage to live up to that moral standard as they wished or even as they think they did. This news might discourage them and might make them give up completely on the cause. Moreover, iSAGE can reveal news that might be distressing at first, such as the fact that one’s sexual orientation is different or wider than initially thought. Thus, it is important to think of how iSAGE should deliver advice and information so that it does not alienate users.

Another set of objections might come from the literature on the “quantified self” or “quantified relationship” (see Danaher et al., 2018a, 2018b). Relevant objections from this literature might include the Measurement-Management Objection (we would only have access to information about ourselves that is ‘measurable’ or at least translatable into terms iSAGE can recognize and process; this may lead us to ‘manage’ only a subset of relevant aspects of ourselves, while ignoring aspects that are harder to quantify); the Instrumental/Intrinsic Value Objection (users might interpret iSAGE as effectively ‘gamifying’ moral development, leading them to view the cultivation of moral character primarily in instrumental terms rather than as a good in itself); and the Neoliberalization Objection (iSAGE might feed into the neoliberal political project, ‘individualizing’ responsibility for moral betterment, possibly to the exclusion of making necessary changes to systemic or structural factors) (as summarized by Danaher et al., 2018a, 2018b).

Although our aim in this piece is not to raise and respond to every potential objection to our iSAGE proposal, we raise these concerns here to prompt further reflection and discussion by bioethicists and others engaged in the AMA debate. For example, someone might want to raise concerns about LLMs overwriting one's authority over one's self, thereby biasing self-development towards a certain direction and possibly realising self-fulfilling prophecies.^1^

Despite these legitimate issues and difficulties, the potential merits of exploring systems like iSAGE become clear when contrasted with other AMA proposals. Unlike exhaustive AMAs that fully outsource moral reasoning to algorithms using fixed frameworks, iSAGE allows individuals to continue developing as opposed to delegating moral reasoning and decision-making capacities. Compared to auxiliary AMAs relying on user-specific inputs which are both static and potentially inauthentic, iSAGE can offer a third-party and longitudinal perspective that track shifts in personality, preferences, and values. And contrasted with Socratic assistants and AI-based nudging systems that assess moral reasoning or provide nudges based on predefined frameworks, iSage offers a flexible, personalized approach based on modelling user’s lived moral experience as expressed in their digital traces. Moreover, iSAGE does all this while retaining general LLM functions that allow for personalized consumption of information, including advice, and for integration with real-world information and factual knowledge bases, while also potentially leveraging respected third party personalized LLMs. To us, these many advantages suggest that we should take the idea of personalized LLM-based self-knowledge and moral enhancement systems seriously. They offer a potentially meaningful way to augment self-knowledge and moral reasoning in a world in which selves and values resist easy description or one-size-fits-all categorization, while also accessing a wider world of moral knowledge and advice.

## Footnotes

1 We thank an anonymous reviewer for this point.
